# Supplementary material for: Nucleosome flipping drives kinetic proofreading and processivity by SWR1
Source: Nature. 2024 Nov 6;636(8041):251–7. doi: 10.1038/s41586-024-08152-y (PMC11618073; doi:10.1038/s41586-024-08152-y)
Supplement: Supplementary file 1 — Uncropped raw gels for Extended Data Figs. 2a, 2b and 4j and legends for Supplementary Videos [file 41586_2024_8152_MOESM1_ESM.pdf]

nature portfolio

https://doi.org/10.1038/s41586-024-08152-y

Supplementary information

Nucleosome flipping drives kinetic proofreading and processivity by SWR1

In the format provided by the authors and unedited

Supplementary Figure 1: Uncropped gels.

Extended Data Fig. 2a

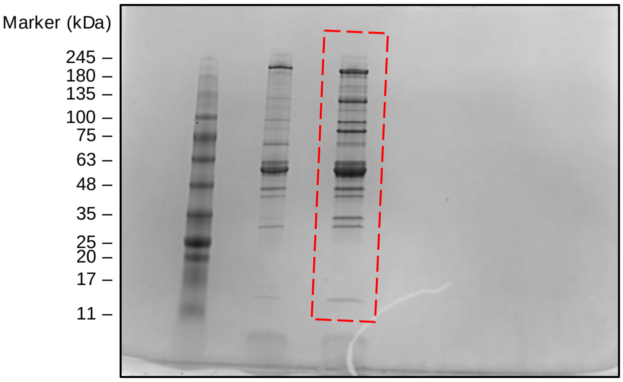

Coomassie scan

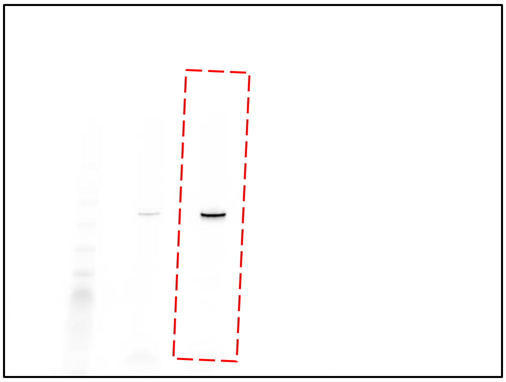

Cy5 scan

Extended Data Fig. 2b

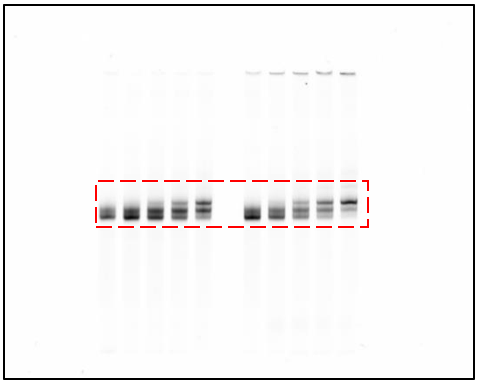

Extended Data Fig. 4j

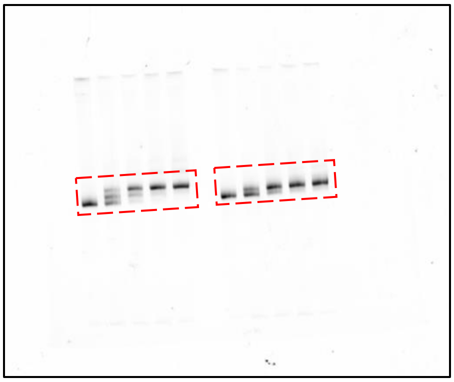

Captions for Supplementary Videos:

Supplementary Video 1. 180° rocking movie of SWR1–nucleosome complex in configuration I coordinates fitted into the 3.8 Å volume. The atomic coordinates are colored as in Extended Data Fig. 7h, and the 3.8 Å volume is colored to match the fitted atomic coordinates.

Supplementary Video 2. 180° rocking movie of SWR1–nucleosome complex in configuration II coordinates fitted into the 4.7 Å volume. The atomic coordinates are colored as in Extended Data Fig. 7i, and the 4.7 Å volume is colored to match the fitted atomic coordinates.

Supplementary Video 3: SWR1-mediated nucleosome flipping. Distal (orange) and proximal (blue) H2A–H2B. Two intermediate coordinates of SWR1–nucleosome flipping were modelled using the 2D classes (Figure 5f and Extended data Fig. 9b). A third modelled intermediate of SWR1–nucleosome complex in configuration I (before DNA at SHL6-7 stabilization by Arp6–Swc6) was built with the nucleosome coordinates inverted, and DNA matching that in configuration II. Two morphs were generated: First between configuration II and the first modelled coordinates. Second, between the first modelled coordinates, followed by the second modelled coordinates and ending with the third modelled configuration I. Morphs were spliced to generate the summary movie.
