# Supplementary material for: Nucleosome flipping drives kinetic proofreading and processivity by SWR1
Source: Nature. 2024 Nov 6;636(8041):251–7. doi: 10.1038/s41586-024-08152-y (PMC11618073; doi:10.1038/s41586-024-08152-y)
Supplement: Supplementary file 2 — Reporting Summary [file 41586_2024_8152_MOESM2_ESM.pdf]

nature portfolio

Corresponding author(s): Dale B Wigley, David S Rueda

Last updated by author(s): Aug 24, 2024

Reporting Summary

Nature Portfolio wishes to improve the reproducibility of the work that we publish. This form provides structure for consistency and transparency in reporting. For further information on Nature Portfolio policies, see our Editorial Policies and the Editorial Policy Checklist.

Statistics

For all statistical analyses, confirm that the following items are present in the figure legend, table legend, main text, or Methods section.

n/a	Confirmed	
☐	☒	The exact sample size (*n*) for each experimental group/condition, given as a discrete number and unit of measurement
☐	☒	A statement on whether measurements were taken from distinct samples or whether the same sample was measured repeatedly
☒	☐	The statistical test(s) used AND whether they are one- or two-sided<br>*Only common tests should be described solely by name; describe more complex techniques in the Methods section.*
☒	☐	A description of all covariates tested
☒	☐	A description of any assumptions or corrections, such as tests of normality and adjustment for multiple comparisons
☐	☒	A full description of the statistical parameters including central tendency (e.g. means) or other basic estimates (e.g. regression coefficient) AND variation (e.g. standard deviation) or associated estimates of uncertainty (e.g. confidence intervals)
☒	☐	For null hypothesis testing, the test statistic (e.g. *F*, *t*, *r*) with confidence intervals, effect sizes, degrees of freedom and *P* value noted<br>*Give P values as exact values whenever suitable.*
☐	☒	For Bayesian analysis, information on the choice of priors and Markov chain Monte Carlo settings
☒	☐	For hierarchical and complex designs, identification of the appropriate level for tests and full reporting of outcomes
☒	☐	Estimates of effect sizes (e.g. Cohen's *d*, Pearson's *r*), indicating how they were calculated

Our web collection on statistics for biologists contains articles on many of the points above.

Software and code

Policy information about availability of computer code

Data collection	Single-molecule videos were acquired using a home-built LabView scripts or HCImage 4.6.1.3 (Hammamatsu). CryoEm data was collected on a Titan Krios microscope.
Data analysis	Single-molecule data extraction: IDL 8.4. Single-molecule data viewing: MATLAB R2022b. Single-molecule HMM analysis: vbFRET or tMAVEN 0.2.0. Single-molecule data plotting: Igor Pro 8.04. All custom analysis scripts used can be found on the groups github page: (https://github.com/singlemoleculegroup). CryoEM data processing: MotionCor2, CTFFIND4, cryoSPARC 3.3.2, RELION 4.0, PHENIX 1.20.1, UCSF Chimera 1.16, COOT 0.9.8.3 , ChimeraX 1.6.1, AlphaFold 2.1.0

For manuscripts utilizing custom algorithms or software that are central to the research but not yet described in published literature, software must be made available to editors and reviewers. We strongly encourage code deposition in a community repository (e.g. GitHub). See the Nature Portfolio guidelines for submitting code & software for further information.

Data

Policy information about availability of data

All manuscripts must include a data availability statement. This statement should provide the following information, where applicable:

- Accession codes, unique identifiers, or web links for publicly available datasets
- A description of any restrictions on data availability
- For clinical datasets or third party data, please ensure that the statement adheres to our policy

Electron density maps are deposited at the Electron Microscopy Database (accession codes EMDB-18471 & EMDB-18472) and atomic coordinates are deposited at the Protein Databank (PDB ID codes 8QKU & 8QKV). Initial models used for model building include PDB ID:6GEN & 6GEJ, as well as an AlphaFold generated model of Swc2. The datasets generated during and/or analysed during the current study will be available from the corresponding author on reasonable request.

Research involving human participants, their data, or biological material

Policy information about studies with human participants or human data. See also policy information about sex, gender (identity/presentation), and sexual orientation and race, ethnicity and racism.

Reporting on sex and gender	N/A
Reporting on race, ethnicity, or other socially relevant groupings	N/A
Population characteristics	N/A
Recruitment	N/A
Ethics oversight	N/A

Note that full information on the approval of the study protocol must also be provided in the manuscript.

Field-specific reporting

Please select the one below that is the best fit for your research. If you are not sure, read the appropriate sections before making your selection.

☒ Life sciences ☐ Behavioural & social sciences ☐ Ecological, evolutionary & environmental sciences

For a reference copy of the document with all sections, see nature.com/documents/nr-reporting-summary-flat.pdf

Life sciences study design

All studies must disclose on these points even when the disclosure is negative.

Sample size	No prior sample size calculation was performed. Sample sizes were selected based on previous experience. All observations were made on sufficient numbers of individual molecules, when possible more than 100. For structural determination, the number of micrographs in our cryoEM data collection was chosen accordingly to obtain the required resolution.
Data exclusions	Inclusion criteria for single-molecule data are detailed in the Methods section.
Replication	Single-molecule data was independently replicated at least twice. The total number of traces used for each dataset are indicated on each figure. For bulk assays (gels) two independant repeats were performed one of which is shown, attempts at replication were successful.
Randomization	In the Fourier shell correlation (FSC) measurement in RELION 4.0 pipeline, data from the Refine3D job was randomly divided into two halves resulting in two independently determined 3D volumes that were used for the FSC calculation through a Postprocess job.
Blinding	Blinding was not relevant to the experiments in this study. Cryo-EM and biochemcial data were collected and processed in an unbiased manner.

Reporting for specific materials, systems and methods

We require information from authors about some types of materials, experimental systems and methods used in many studies. Here, indicate whether each material, system or method listed is relevant to your study. If you are not sure if a list item applies to your research, read the appropriate section before selecting a response.

Materials & experimental systems

n/a	Involved in the study
☒	☐ Antibodies
☐	☒ Eukaryotic cell lines
☒	☐ Palaeontology and archaeology
☒	☐ Animals and other organisms
☒	☐ Clinical data
☒	☐ Dual use research of concern
☒	☐ Plants

Methods

n/a	Involved in the study
☒	☐ ChIP-seq
☒	☐ Flow cytometry
☒	☐ MRI-based neuroimaging

Eukaryotic cell lines

Policy information about cell lines and Sex and Gender in Research

Cell line source(s)	Spodoptera frugiperda Sf9, Thermo Fisher Scientific, 11496015<br>Trichoplusia ni High Five,Thermo Fisher Scientific, B85502
Authentication	Cell lines not authenticated
Mycoplasma contamination	Cell lines were not tested for mycoplasma
Commonly misidentified lines<br>(See ICLAC register)	None used

Plants

Seed stocks	*Report on the source of all seed stocks or other plant material used. If applicable, state the seed stock centre and catalogue number. If plant specimens were collected from the field, describe the collection location, date and sampling procedures.*
Novel plant genotypes	*Describe the methods by which all novel plant genotypes were produced. This includes those generated by transgenic approaches, gene editing, chemical/radiation-based mutagenesis and hybridization. For transgenic lines, describe the transformation method, the number of independent lines analyzed and the generation upon which experiments were performed. For gene-edited lines, describe the editor used, the endogenous sequence targeted for editing, the targeting guide RNA sequence (if applicable) and how the editor was applied.*
Authentication	*Describe any authentication procedures for each seed stock used or novel genotype generated. Describe any experiments used to assess the effect of a mutation and, where applicable, how potential secondary effects (e.g. second site T-DNA insertions, mosiacism, off-target gene editing) were examined.*
